# Growth hormone biases amygdala network activation after fear learning

**DOI:** 10.1038/tp.2016.203

**Published:** 2016-11-29

**Authors:** B Gisabella, S Farah, X Peng, A Burgos-Robles, S H Lim, K A Goosens

**Affiliations:** 1 McGovern Institute for Brain Research, Department of Brain and Cognitive Sciences, Massachusetts Institute of Technology, Cambridge, MA, USA

## Abstract

Prolonged stress exposure is a risk factor for developing posttraumatic stress disorder, a disorder characterized by the ‘over-encoding' of a traumatic experience. A potential mechanism by which this occurs is through upregulation of growth hormone (GH) in the amygdala. Here we test the hypotheses that GH promotes the over-encoding of fearful memories by increasing the number of neurons activated during memory encoding and biasing the allocation of neuronal activation, one aspect of the process by which neurons compete to encode memories, to favor neurons that have stronger inputs. Viral overexpression of GH in the amygdala increased the number of amygdala cells activated by fear memory formation. GH-overexpressing cells were especially biased to express the immediate early gene c-Fos after fear conditioning, revealing strong autocrine actions of GH in the amygdala. In addition, we observed dramatically enhanced dendritic spine density in GH-overexpressing neurons. These data elucidate a previously unrecognized autocrine role for GH in the regulation of amygdala neuron function and identify specific mechanisms by which chronic stress, by enhancing GH in the amygdala, may predispose an individual to excessive fear memory formation.

## Introduction

Memories are encoded by sparse neuronal networks,^1^ yet it is clear that many more cells are eligible to participate in an associative memory trace, for example, by receiving the appropriate sensory inputs, than actually are recruited.^2, 3, 4^ It has been suggested that the low rate of participation in memory encoding is the result of local competition within the neuronal network (a process referred to as memory ‘allocation'), and that excitability, regulated by the transcription factor CREB, is especially important for determining the ‘winners' of the allocation process.^5, 6, 7, 8, 9, 10, 11, 12^ It is also thought that the number of neurons that encode a memory is a stable property of the network.^3, 13^ However, the size and allocation of memory has not been examined under conditions of pathological memory strength.

A protracted history of stress exposure before trauma is a risk factor for excessive encoding of the traumatic memory in both humans^14, 15, 16^ and rodents.^17^ Studies have shown that growth hormone (GH) synthesized in the amygdala is approximately doubled by chronic stress,^17^ and both chronic stress^18^ and high levels of GH in the basolateral complex of the amygdala (BLA) are sufficient to increase the strength of fear memories,^17^ suggesting a novel molecular link between prolonged stress exposure and the over-encoding of a traumatic memory. Virtually nothing is known about the role of GH within the amygdala, but it is possible that GH could promote the over-encoding of traumatic memories by dysregulating the recruitment and allocation of neuronal activity during fear learning.

Using viral overexpression of GH in the amygdala to mimic the enhanced expression of GH in this brain region observed after chronic stress, we determined whether excessive GH in the amygdala impacted the recruitment and distribution of neuronal activity across individual neurons after fear learning. We found that GH overexpression led to a significant increase in the number of cells expressing the immediate early gene (IEG) c-Fos in the lateral (LA) and basolateral (BL) nuclei of the amygdala after fear conditioning, and that GH-overexpressing cells were disproportionately more likely to express c-Fos than either uninfected neighbor cells or cells in the contralateral amygdala expressing only green florescent protein (GFP). However, the enhanced recruitment of cells during fear memory acquisition arose from increased c-Fos in both GH-overexpressing cells and their proximal neighbors. We also found that high levels of GH dramatically enhanced spine density in pyramidal neurons of LA and BL. Thus, like CREB,^5, 6, 7, 8, 9, 10, 11, 12^ GH is a ‘memory attractor' and biases the allocation of neuronal activity through autocrine actions. However, in contrast with CREB, GH also increases the number of neurons that are activated following fear conditioning through paracrine mechanisms. We believe these results provide the first direct evidence that locally synthesized GH regulates neuronal morphology and function in the adult amygdala, and suggest a specific set of novel mechanisms by which chronic stress, which increases GH within the amygdala,^17^ is a risk factor for the over-encoding of traumatic memories.^14, 19^

## Materials and methods

Additional details are provided in the Supplementary Materials and Methods.

### Experimental subjects

All experiments used adult male Long–Evans rats (250–350 g, Charles River, Raleigh, NC, USA) and were approved by both the Institutional Animal Care and Use Committee of the Massachusetts Institute of Technology and the Animal Care and Use Review Office of the US Army Medical Research and Materiel Command.

### Virus

Herpes simplex viral (HSV) amplicons were used to overexpress either GH and GFP, or GFP alone. The viruses were as previously described.^17^

### Stereotaxic surgery

Anesthetized rats received intra-BLA injections of HSV amplicons to express GH and GFP (the experimental hemisphere) or only GFP (the control hemisphere). Each rat received two infusions of virus into the BLA of each hemisphere (1 μl: anterior/posterior −2.4, medial/lateral ±5.1, dorsal/ventral −7.2; and 1 μl: anterior/posterior −2.4, medial/lateral ±5.1, dorsal/ventral −6.9) for a total of 2 μl per BLA.

### Fear conditioning

To investigate the impact of GH overexpression on fear acquisition, rats (*n*=7–8/group) received stereotaxic delivery of HSV amplicons as described above. After six days of recovery, each rat was placed in a fear conditioning box (MedAssociates, St. Albans, VT, USA) within a larger, sound-attenuating chamber. Rats were placed in the chamber for three minutes before four tone (10 s, 85dB, 10kHz) – footshock (2 s, 0.8 mA) pairings were administered (60–200 s variable ITI). The following day, rats were returned to the context for an extinction session (10 min). The following day, rats were placed in a novel context for auditory fear extinction (3 min pre; 60 s, 85dB, 10kHz tone; 60 s post-tone period).

To investigate the impact of GH overexpression on fear extinction, rats received auditory fear conditioning in a novel context. Three minutes after placement in the box, rats received five tone (15 s, 85dB, 2kHz)-footshock (1 s, 0.5 mA) pairings (co-terminating; 60 s ITI). Two days later, rats received stereotaxic delivery of HSV amplicons as described above (*n*=4/group). After six days of recovery, rats were placed in the same context for context extinction training (15 min). Three hours later, rats were placed in a novel context for two minutes before 15 tone presentations were given (15s, 85dB, 2kHz, 60 s ITI). The next day, contextual (15 min) and auditory extinction recall (2 min pre; 15s, 85dB, 2kHz, 60 s ITI) were tested in sessions separated by three hours.

To investigate the size and allocation of fear memories, rats received stereotaxic delivery of HSV amplicons in the BLA as described above. After six days for recovery and transgene expression, animals underwent auditory fear conditioning in a novel context. Rats in the Paired condition (*n*=7) received 3 tone-footshock pairings. Such training produces a reliable and robust long-term fear memory for the environmental context and the tone. Rats in the Unpaired condition (*n*=5) received pseudorandom, non-overlapping presentations of 3 tones and 3 footshocks. This training produces a long-term fear memory for the environmental context, but produces much weaker long-term associative fear memory for the tone.^3, 20, 21, 22, 23^ On the morning of the experiment, all rats were transported in their home cages from the vivarium to a holding room in which no behavioral testing was conducted. This transport occurred at least one hour prior to the onset of behavioral training. All behavioral experiments were conducted between 1000 h and 1300 h. All rats were placed in the conditioning chambers for three minutes before three tones were delivered (20 s, 85 dB, 2kHz, 120 s ISI). For rats in the Paired condition, each tone co-terminated with a footshock (2 s, 0.4 mA). For rats in the Unpaired condition, shocks were delivered in between tones (90, 30 and 58 s after tone offset). Rats remained in the box for one minute after the final tone before being removed. During fear conditioning, room lights and overhead box lights were on, acetic acid (1%) was used to scent and clean the boxes, and rats were transported in white plastic shoe boxes.

### Tissue processing and immunohistochemistry

#### For GH and NeuN Immunohistochemistry

A naïve rat was perfused with 0.1M PBS and 4% PFA. The brain was cryoprotected in 30% sucrose in 0.1M PBS and cryosectioned into 40-mm thick sections. Sections were washed in 0.01M PBS with 0.2% Triton-X, followed by a 1 h incubation in 2% BSA at room temperature, and incubated in primary antibodies anti-GH (rabbit, 1:200, NIH), anti-NeuN (mouse, 1:200, mab377) in 4°C overnight, and secondary antibodies Alexa Fluor 488 Goat Anti Rabbit and 594 Goat Anti Mouse (Invitrogen) at room temperature for 2 h.

#### For GH and MAP2 immunocytochemistry

Amygdala neurons were fixed after 28 days *in vitro* in 4% paraformaldehyde, 4% sucrose in phosphate-PBS, pH 7.4, for 5 min, permeabilized with cold 0.25% Triton X-100 for 5 min, and blocked in 5% normal goat serum (Invitrogen) for 1 h at room temperature (RT). Immunostaining was performed by incubating with the primary antibodies (rabbit anti-GH, National Hormone and Peptide Program, 1:200; chicken anti-MAP2, cat# ab5392, Abcam, 1:5000) at 4°C overnight, followed by incubation with the secondary antibodies Alexa Fluor 488 Goat Anti Rabbit (Invitrogen) and DyLight647 Donkey Anti Chicken (Jackson ImmunoResearch) at room temperature for 45 min.

#### For c-Fos and GFP dual immunohistochemistry

Rats underwent fear conditioning (Paired or Unpaired) six days after HSV injections. One hour after fear conditioning, animals were perfused with 0.1M PBS and 4% PFA. Brains were then cryoprotected in 30% sucrose in 0.1M PBS and cryosectioned into 40-mm thick sections (4°C for one week). Sections with moderate levels of infection were chosen for immunohistochemistry because high levels of infection produced saturated fluorescence, making it difficult to confirm GFP expression in discrete cell bodies. Slices were defined as having moderate level of infection when cytoarchitecture was clearly distinguishable and virus infection area covered the majority of the BLA with no spread outside of this region. Slices were defined as having high level of infection when GFP expression was saturated, resulting in reduction of clarity of cytoarchitecture and virus spread outside of the BLA was significant. Levels of infection were determined by an experimenter blinded to experimental group and to behavioral data.

Three sections containing the BLA were selected from each rat (*n*=7 in the Paired condition and *n*=5 in the Unpaired condition). Sections were matched for rostral-caudal position of the BLA across rats. Sections were washed in 0.01M PBS with 0.2% Triton-X, followed by 1 hr incubation in 2% BSA at room temperature, and incubated in primary antibodies (rabbit anti-c-Fos antibody, cat# SC-253, lot# A2313, Santa Cruz Biotechnology, 1:1000; chicken anti-GFP antibody, cat# A10262, lot# 1296691, Invitrogen, 1:1000) at 4º C on a rocking table for 48 h. Sections were then washed in 0.01M PBS with 0.2% Triton-X and incubated in Alexa Fluor secondary antibodies (goat anti-chicken Alexa Fluor 488, cat# A11039, lot# 1599396, 1:300; and goat anti-rabbit Alexa Fluor 594, cat# A11037, lot# 1558726, 1:300, Invitrogen) for 3 h at room temperature on a rocking table, washed with 0.01M PBS, and mounted on gelatin-coated glass slides. Sections were counterstained with DAPI and coverslipped with Vectashield anti-fade mounting media (Vector Laboratories, Burlingame, CA, USA).

### For dendritic spine analysis

Rats (*n*=7) were perfused with 0.1M PBS and 4% PFA six days after viral infusion. Brains were cryoprotected in 30% sucrose in 0.1M PBS (pH 7.4) and cryosectioned into coronal 40 mm sections containing the BLA. Sections were mounted on gelatin coated slides to quantify dendritic spine density from images captured using confocal microscopy.

#### Amygdala cell cultures

The protocol for generating amygdala neuronal cultures was adapted from protocols for hippocampal neuron cultures.^24, 25^ Briefly, rat amygdala was dissected from E19 embryos and placed in Calcium-free Hank's Balanced Salt Solution (HBSS; Invitrogen) containing 1% papain for 20 min, triturated in Basal Media Eagle (Invitrogen) supplemented with B-27 (Invitrogen), and plated at 100,000 cells per milliliter in Neural Basal (NB) (Invitrogen) supplemented B-27, 1% penicillin and streptomycin (Invitrogen), and 1% glutaMAX (Invitrogen) on poly-D-lysine-coated (Sigma-Aldrich) coverslips in 24-well plates. Cells were maintained at 37°C, 5% CO_2_, 95% humidity. The medium was partially changed weekly.

### *In situ* hybridization

Fluorescence *in situ* hybridization staining was carried out using the QuantiGene ViewRNA ISH Tissue 2-Plex kit (Affymetrix, Santa Clara, CA, USA) and the staining procedures were performed according to the manufacturer's protocol.

### Microscopy and data analyses

#### For dendritic spine quantification

Confocal microscopy images were analyzed using Neurolucida software with Autospine (Neurolucida version 10) to measure spine density in apical branches of BL neurons using an approach previously described for the BLA.^26, 27, 28^

#### For *in situ* hybridization

Fluorescent microscopy images were acquired using Zeiss AxioVision software (Carl Zeiss MicroImaging, Thornwood, NY, USA) interfaced with a Zeiss Axio Observer A1 fluorescence microscope with an AxioCam MRm camera. Images were all captured using a 63 × objective from amygdala nuclei using documented landmarks.^29^ Two or three images (142 μm X 106.5 μm) were captured for each region from each section (5-6 sections matched for rostral- caudal position across the amygdala from three rats). Images were exported as 8-bit TIFF files and nuclear expression of target mRNAs was quantified using ImageJ software (NIH, Bethesda, MD, USA) by at least two blinded observers. The total number of all cells (determined by quantifying DAPI+ nuclei) was calculated for each image. The expression of target mRNAs was quantified by examining the expression of fluorescent grains immediately adjacent or overlapping with each nucleus. GH was identified as red grains (using a CY3/TRITC filter); GAD67-expressing cells were identified by green grains (CY5 filter, green). The number of cells expressing both GH and GAD67 was also identified.

#### For dual-immunohistochemistry quantification

C-Fos was quantified by confocal imaging followed by manual counting for individual markers using ImageJ. An average of 15-20 images of the LA and BL were captured per rat using a Zeiss Axio Imager Z2 confocal microscope with a 40x objective lens. ZEN software was used to acquire fluorescent images of Z stacks of 144.5mm x 144.5mm x 2.2 mm (length x width x depth).

### Statistics

The number of animals (*n*) in the experimental and control groups for each experiment are reported in the figure legends. For all statistical tests, the significance threshold was *P*<0.05.

The statistical significance was analyzed using ANOVA (analysis of variance) or one-tailed, one sample *t*-tests, as indicated in the 'Results' section. The *post hoc* Fisher's PLSD tests were performed after a significant omnibus F-ratio. The factors used in the ANOVAs included viral infusion (GFP and GH), cell population (infected and uninfected), region (LA and BL), conditioning (paired and unpaired) and spine type (thin, mushroom and stubby). One-way ANOVA was used for the analysis of single factors, and two-way ANOVA was used for the analyses of two factors.

## Results

### GH produced by excitatory BLA neurons enhances long-term fear memory

**Figure 1:**
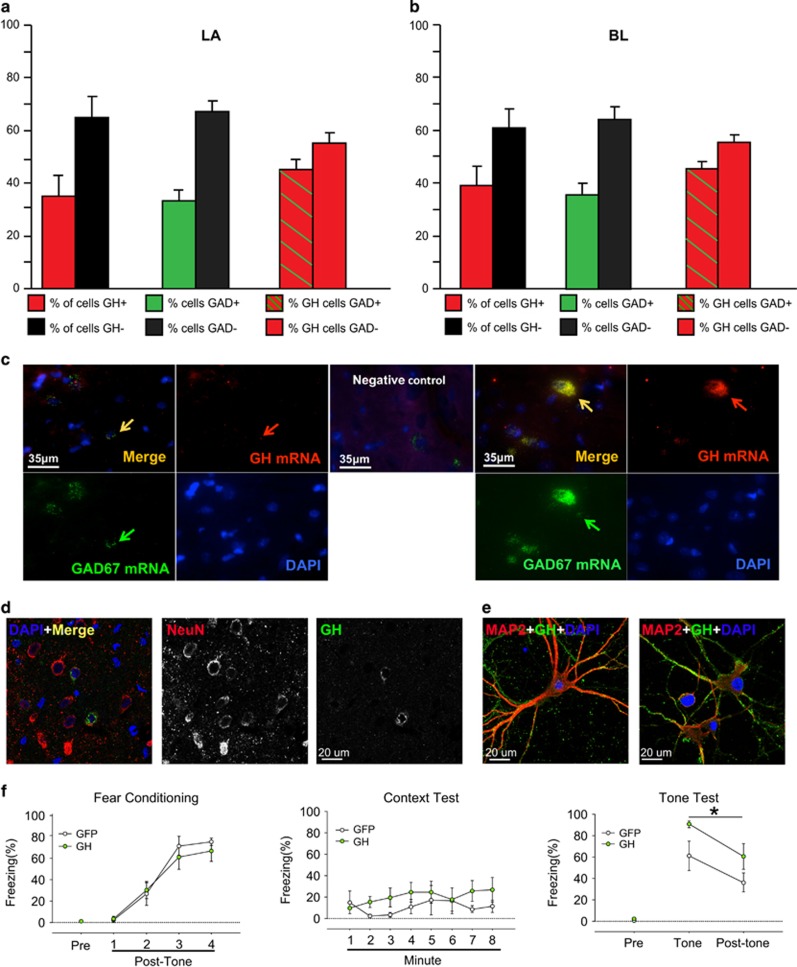
Growth hormone is expressed in excitatory and inhibitory cells in the amygdala. Double *in situ* hybridization against growth hormone (GH; red) and GAD67 (green), a marker of inhibitory interneurons, was conducted in the rat amygdala to quantify nuclear expression of these transcripts. (**a**) Top: the bar graphs display the overall percentage of GH+ cells (left), the overall percentage of GAD67+ cells (middle) and the percentage of GH+ cells that are GAD67+ (right) in the lateral nucleus of amygdala (LA). Bottom: representative double-positive cells from the LA are shown. Blue signal represents nuclear DAPI staining (*n*=1340). Red signal indicates GH mRNA (*n*=617), green signal indicates GAD67 mRNA (*n*=535) and yellow signal corresponds to overlap of GH and GAD67 mRNA. (**b**) Top: bar graphs show the percentages of different cell populations in the basolateral nucleus (BL), as shown in **a** (Top). Bottom: representative double-positive cells from the BL. Blue signal represents nuclear DAPI staining (*n*=1341). Red arrows indicate representative nuclei containing only GH mRNA (*n*=624), green arrows indicate representative nuclei containing only GAD67 mRNA (*n*=493) and yellow arrows signify representative nuclei containing both GH and GAD67 mRNA. The bar graphs reveal that GH mRNA is broadly expressed across LA and BL (35–40% of nuclei). Scale bars represent 35 μm. The '*n*' reported represent the total number of cells counted across rats (three rats). (**c**) For some brain sections containing LA and BL, *in situ* hybridization was performed without adding the probe to target GH (negative control); all other steps were identical to those used for other sections. This enabled visualization of background staining. We observed no red puncta for the GH signal with this method. Some diffuse red haze was present, indicating nonspecific binding. Thus, the discrete red puncta observed in **a** and **b** represent true, albeit sparse, signal. (**d**) Coronal sections of the rat basolateral complex of the amygdala (BLA) were stained for GH protein (green), the neuronal marker NeuN (red), and the nuclear marker DAPI (blue). A colorized merged image of a single slice in the *z* axis is shown (left; × 63). The individual channels for GH and NeuN are also depicted (middle, right). GH is clearly expressed by excitatory pyramidal neurons in the BLA. Scale bar indicates 20 μm. (**e**) Primary cell cultures of amygdala were generated and stained for GH (green), mitogen-activated protein kinase (MAPK; red) and DAPI (blue). GH is strongly expressed in the dendrites and cell bodies of excitatory pyramidal amygdala neurons ( × 63). Scale bars represent 20 μm. (**f**) GH and GFP (GH group) or GFP only (GFP group) was overexpressed in the BLA before auditory fear conditioning. Conditional freezing during fear conditioning, context extinction and tone extinction are depicted. All data shown are means±s.e.m. GFP, green florescent protein.

Although prolonged stress increases GH in the BLA (consisting of the LA and BL), a subregion of the amygdala that is especially important for the acquisition and storage of traumatic memories,^30^ it is not known what type of neurons in the amygdala produce GH. We sought to determine the breadth of expression of GH across the BLA by using double fluorescent *in situ* hybridization against GH messenger RNA (mRNA) and GAD67 mRNA, the predominant marker for inhibitory interneurons in this region.^31, 32^ This quantification (Figures 1a and b) revealed that a significant portion of cells in the LA and BL express nuclear GH (35–40%), with ∼56% of these GH+ cells lacking the GABAergic interneuron marker GAD67. Though the GH signal was sparse, it represents true signal; a negative control run without GH probe revealed the complete absence of red puncta (Figure 1c). GH mRNA is thus broadly expressed in cells in the LA and BL nuclei, including inhibitory interneurons. These data cannot rule out the possibility that non-neuronal cells may also express GH.

To further examine the expression of GH in excitatory BLA neurons, we performed immunostaining for GH on coronal rat brain sections containing the BLA. We confirmed that individual BLA neurons with pyramidal morphology express GH protein (Figure 1d). We also examined the expression of GH protein in cultured amygdala neurons using immunocytochemistry. GH protein was strongly expressed in the dendrites and cell bodies of excitatory pyramidal neurons (Figure 1e).

To investigate mechanisms of allocation of neuronal activity under pathological conditions, we sought to overexpress GH in the BLA. We used an HSV amplicon with a bicistronic viral promoter for broad bilateral expression across excitatory pyramidal neurons^32^ of either rat GH and enhanced GFP (‘GH' group) or GFP alone (‘GFP' group) in the BLA for 6 days before auditory fear conditioning (Figure 1f). This amplicon system preferentially transduces excitatory neurons^31^ and is virtually identical to that used to investigate the role of CREB in neuronal recruitment and allocation during fear learning.^3, 6, 33^ Also, 6 days is approximately the number of days that a stressor must be repeated to increase fear memory acquisition.^17^

Rats with elevated levels of GH in the BLA during auditory fear conditioning displayed significantly higher long-term auditory fear memories compared with rats with only GFP in the BLA (Figure 1f; tone test, main effect of viral infusion: F(1,13)=4.74, *P*=0.04), replicating an effect we reported previously.^17^ In contrast, overexpression of GH in the BLA of rats after auditory fear conditioning had no impact on subsequent auditory fear memory recall or extinction (Supplementary Figure 1 and Supplementary Results). These results suggest that GH in the BLA is more important for fear memory formation than fear memory retrieval or performance.

### GH overexpression alters the allocation of neuronal activity in the BLA in response to fear conditioning

**Figure 2:**
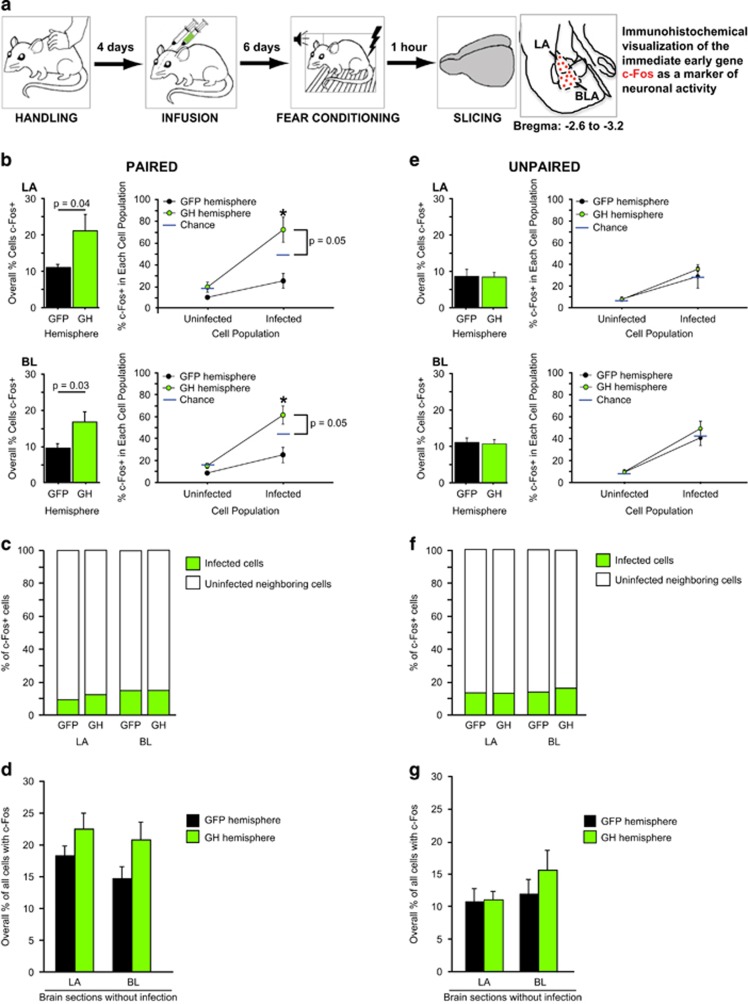
Growth hormone (GH) overexpression increases the number of cells activated by fear learning and biases the allocation of neuronal activity. (**a**) Schematic diagram depicting timeline of experimental procedures. The animals were handled every day for 4 consecutive days before stereotaxic surgery. A herpes simplex viral (HSV) amplicon to co-express GH and green florescent protein (GFP) was infused into the right amygdala, and an HSV amplicon to express GFP alone was infused into the left amygdala of each rat. After 6 days for recovery, the animals received paired (*n*=7) or unpaired (*n*=5) auditory fear conditioning. All the rats were killed for immunohistochemistry 1 h after fear conditioning. Depicted coronal brain section is adapted from ref. 29. (**b**) Left panels: the number of cells encoding a fear memory, as measured by the overall percentage of cells showing c-Fos expression after paired fear conditioning, is shown. The cells in the GH-overexpressing hemisphere were 1.90 and 1.76 times more likely to be c-Fos+ than cells in the GFP-overexpressing hemisphere in the lateral (LA) and basal (BL) amygdala, respectively. These numbers were used to calculate the anticipated percentage of c-Fos+ cells in the infected and uninfected cell populations of the GH-overexpressing hemisphere, assuming random distribution of c-Fos expression across these populations (‘chance', indicated by blue lines; see the 'Materials and methods' section for details). Right panels: the percentage of the infected and uninfected cells that were c-Fos+ in the GH-overexpressing and GFP-overexpressing hemispheres is shown. GH-overexpressing neurons were significantly more likely to express c-Fos than expected by chance in both LA and BL; this bias was not observed in uninfected neighbor cells. (**c**) These bar plots show the distribution of c-Fos across the infected and uninfected cell populations in the LA and BL for rats after paired fear conditioning. (**d**) The percentage of c-Fos+ cells induced by paired fear conditioning in sections lacking any viral infection or transgene expression is shown. The level of c-Fos expression did not differ in these ‘neighboring' sections from the GH-overexpressing or GFP-overexpressing hemispheres. (**e-g**) All measures are depicted as for **a-c**, but for rats that received unpaired fear conditioning. Neither memory allocation nor the number of neurons recruited during fear conditioning differed between the GH-overexpressing hemisphere and the GFP-overexpressing hemisphere for rats in this condition. For each measure, an average value was computed for the GH hemisphere and a second average was calculated for the GFP hemisphere for each rat. All the statistical tests were computed using these per-animal averages. All the data shown are means±s.e.m. Exact *P*-values are reported in each panel.

GH overexpression in the BLA increased long-term fear memory strength (Figure 1e), and we reasoned that differences in fear memory strength across rats could impact the level and allocation of neuronal activation, hence we sought to examine whether GH overexpression changes the allocation of neuronal activation in the BLA during memory encoding. To do this, we compared the level and allocation of neuronal activation as a within-subject measure in rats that received intra-BLA infusion of the GH-expressing virus into the right BLA and infusion of the GFP-expressing virus into the left BLA. This enabled us to examine the impact of GH overexpression on neuronal recruitment in the amygdala under conditions where fear levels were matched across the GFP and GH conditions. After 6 days of recovery, the animals received either paired or unpaired auditory fear conditioning, and were killed for immunohistochemistry 1 h after fear conditioning (Figure 2a).

We hypothesized that increased GH in the BLA (for example, resulting from chronic stress^17^) leads to enhanced fear memory strength by increasing the number of cells that are activated by fear learning. We also hypothesized that cells with high levels of GH may preferentially encode fear memories. Because GH is able to enhance the formation of associative plasticity,^34^ biased recruitment of these cells during fear memory encoding would provide an additional mechanism by which GH could promote excessively strong fear memories. To test whether high levels of GH dysregulate these aspects of fear memory encoding, we measured expression of the IEG c-Fos, a marker of neuronal activity,^35^ in the LA and BL after paired or unpaired auditory fear conditioning (Figure 2a; Supplementary Figure 2 and Supplementary Results). Unpaired auditory fear conditioning was used as a control because such conditioning does not lead to a strong associative memory between the tone and shock.^36, 37, 38^

We found that cells in LA and BL of the GH-overexpressing hemisphere were significantly more likely to contain c-Fos after paired fear conditioning in comparison with cells in the hemisphere overexpressing only GFP (Figure 2b, left panels; main effect of viral infusion: F(1,9)=5.8, *P*=0.04 for LA; main effect of viral infusion: F(1,8)=6.6, *P*=0.03 for BL). Specifically, the cells in the LA and BL in the GH-overexpressing hemisphere were 1.90 and 1.76 times more likely to be c-Fos+ than cells in the GFP-only hemisphere. This suggests that high levels of GH lead to an increased number of BLA neurons recruited during fear learning.

To determine whether the allocation of neuronal activity was biased by GH overexpression, we used standard methods^3^ and compared the probability that a cell was c-Fos+ in the infected versus uninfected cells of each hemisphere (Figure 2b, right panels). There was significantly higher expression of c-Fos in both the infected and uninfected cells of the GH-overexpressing hemisphere as compared with the GFP-overexpressing hemisphere; this effect was observed in both LA and BL (Figure 2b, right panels, black vs green data points; main effect of viral infusion: F(1,9)=12.15, *P*=0.007 for LA; main effect of viral infusion: F(1,8)=12.24, *P*=0.008 for BL). There was also a significant interaction between hemisphere and cell population: while infected cells expressed more c-Fos than uninfected neighbor cells, this effect was especially pronounced in the GH-overexpressing hemisphere (viral infusion × cell population interaction: F(1,9)=13.72, *P*=0.005 for LA; viral infusion × cell population interaction: F(1,8)=9.3, *P*=0.01 for BL). Here, there was a dramatic difference in the participation of infected versus uninfected cells in the memory trace (~73 versus ~20% for LA and ~62 versus ~15% for BL). However, such an effect could simply arise by chance, as the GH-overexpressing hemisphere had a higher level of c-Fos expression than the GFP-overexpressing hemisphere (Figure 2b, left panels). To account for this, we calculated the expected ‘chance' level of c-Fos expression for both the infected and uninfected cell populations, which assumed that the overall level of c-Fos expression (Figure 2b, left panels) was evenly distributed across infected and uninfected cells (see Supplementary Materials and Methods for details). The probability of a cell expressing c-Fos in the infected cells of LA or BL the GH-overexpressing hemisphere was significantly greater than chance (one-tailed, one sample *t*-test comparing the percentage of infected cells expressing c-Fos within the GH-overexpressing hemisphere to chance; *t*=2.08, *P*=0.05 for LA; *t*=2.14, *P*=0.05 for BL), but this was not observed in the uninfected cells of the same hemisphere (Figure 2b, right panels) (comparing the percentage of uninfected cells expressing c-Fos within the GH-overexpressing hemisphere to chance; *t*=−0.04, *P*=0.52 for LA; *t*=−0.07, *P*=0.52 for BL). These results show that GH-overexpressing cells in the BLA are disproportionately biased to be activated by fear learning, suggesting that GH overexpression alters the allocation of neuronal activity through autocrine mechanisms.

It is of interest to determine whether the increased expression of c-Fos in the GH-overexpressing hemisphere is selectively attributable to increased recruitment of GH-overexpressing cells. To examine this, we computed the percentage of c-Fos expressing cells that were infected and uninfected in the LA and BL of each hemisphere. The percentage of c-Fos expression attributable to the GH-overexpressing cells was similarly low (~13%) across all conditions (Figure 2c; green bars). This suggests that the increased expression of c-Fos in the GH-overexpressing hemisphere is largely due to a modest, yet significant, increase in c-Fos expression in uninfected cells, which represented a large proportion of the cell population examined, as well as a large increase in the probability of c-Fos expression by GH-overexpressing neurons, which represented a small proportion of the cell population examined. Collectively, our results show that GH acts in a paracrine manner to enhance the recruitment of neighboring cells into a fear memory trace, as well as an autocrine manner.

To further examine the paracrine effects of GH on cellular recruitment to a fear memory trace, we calculated the percentage of c-Fos expressing cells in the LA and BL of coronal brain sections near the site of viral infusion, but lacking any expression of GFP in the cell bodies or processes. In this case, there was no significant difference in the c-Fos expression across the GH-overexpressing and the GFP-overexpressing hemispheres (Figure 2d; main effect of viral infusion: F(1,20)=0.15, *P*=0.70; main effect of region: F(1,20)=1.04, *P*=0.32; viral infusion × region interaction, F(1,20)=0.14, *P*=0.71). This suggests that the paracrine effects of GH only occur on cells that lie in physical proximity to a GH-overexpressing cell.

The results from the unpaired control group were dramatically different from the paired group (Figure 2b vs e, left: interaction of infusion × conditioning: F(1,32)=8.36, *P*=0.007; main effect of region: F(1,32)=0.06, *P*=0.81; right: interaction of infusion × conditioning: F(1,32)=14.81, *P*=0.0005; main effect of region: F(1,32)=0.14, *P*=0.71). In the unpaired group, the overall number of c-Fos+ cells was equally low across both the infected cells and the uninfected neighbor cells in both LA and BL (Figure 2e, left panels; main effect of viral infusion: F(1,8)=0.001, *P*=0.97 for LA; main effect of viral infusion: F(1,9)=0.23, *P*=0.82 for BL). There was also no preferential expression of c-Fos in the GH-overexpressing cells in either LA or BL (Figure 2e, right panels; main effect of viral infusion: F(1,7)=0.60, *P*=0.60, viral infusion × cell population interaction: F(1,7)=0.29, *P*=0.61 for LA; main effect of viral infusion: F(1,8)=0.73, *P*=0.42, viral infusion × cell population interaction: F(1,8)=0.69, *P*=0.43 for BL). As was observed for the paired group, c-Fos was largely expressed in uninfected cells, regardless of which the virus was infused into the hemisphere (Figure 2f), and no paracrine effects of GH overexpression were observed in the sections lacking infection (Figure 2g; main effect of viral infusion: F(1,19)=0.87, *P*=0.37; main effect of region: F(1,19)=1.72, *P*=0.21; viral infusion × region interaction, F(1,19)=0.63, *P*=0.44).

### GH increases dendritic spine density in the BLA

Neuronal excitability is believed to exert a profound influence over the ability of individual cells to participate in a fear memory trace.^8, 31, 39^ Dendritic spines represent one important factor controlling the sensitivity of a neuron to input,^40^ and enhanced spine density is one mechanism by which neurons can be preferentially recruited into a memory.^5^ Because GH has a broad role in cell growth outside as well as inside of the brain^41, 42, 43^ and neuronal morphology of the amygdala is regulated by many factors, including stress,^44^ we hypothesized that locally synthesized GH may have an important role in regulating spine density.

**Figure 3:**
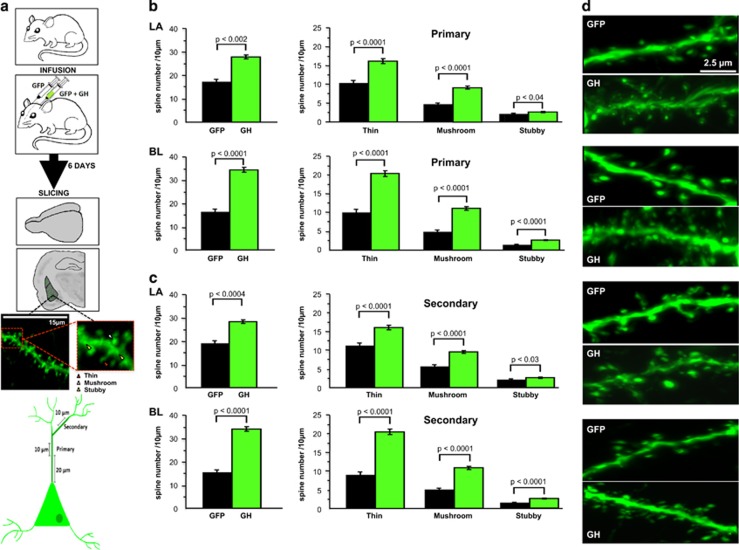
Growth hormone (GH) overexpression results in enhanced density of primary and secondary dendritic spines in the amygdala. (**a**) Upper image: schematic diagram of a neuron depicting the locations where confocal images were taken for quantification. Images consisting of 10 μm of dendritic branch length were taken from primary branches of pyramidal neurons at least 20 μm from the cell body, and images of 10 μm branches were taken from dendrites branching off the primary branches as secondary branches. Lower images: the experimental design shows adult male Long–Evans rats received intra-BLA infusions of herpes simplex viral (HSV) amplicons to overexpress GH (right hemisphere) or green florescent protein (GFP; left hemisphere). The rats were killed 6 days after viral injection, and confocal microscopy images were analyzed using Neurolucida software to quantify spine density in apical branches of LA and BL neurons. (**b**) Infusion of HSV amplicons to overexpress rodent GH (*n*=7 rats) in the LA and BL nuclei resulted in significantly increased density of three types of dendritic spines (mushroom, thin and stubby) relative to spine densities within the hemisphere expressing GFP virus (*n*=6 rats). (**c**) Similar changes were observed in the secondary branches (*n*=6 rats). (**d**) Representative confocal microscopy images depict primary dendritic branches with increased spines from GH-overexpressing (GH) neurons and neurons expressing GFP only in the LA and BL. Scale bar represents 2.5 μm. For each measure, an average value was computed for the GH hemisphere and a second average was calculated for the GFP hemisphere for each animal. All statistical tests were computed using these per-animal averages. All data shown are means±s.e.m. Exact *P*-values are reported in each panel. BLA, basolateral complex of the amygdala; BL, basolateral; LA, lateral.

As in the previous experiment (Figure 2a), we used an HSV amplicon to overexpress (for 6 days) GH and GFP in one BLA and GFP alone in the contralateral BLA of adult rats (Figure 3a). No fear conditioning was administered to these rats. Rather, the rats were killed for spine analysis 6 days after surgery. The dendritic spine density was measured within the apical branches of LA and BL neurons, corresponding to a portion of the dendritic tree of BLA neurons where enhanced spine density is observed following chronic stress.^44^ The spine density was calculated for several subtypes of spines believed to have different functional roles: thin, mushroom and stubby spines.

We found that GH overexpression results in significant increases in the density of all three types of dendritic spines (mushroom, thin and stubby) in the primary branches of LA and BL pyramidal neurons in comparison with spine densities quantified in the hemisphere expressing the control GFP virus (Figure 3b; left upper panel, main effect of viral infusion, F(1,12)=14.6, *P*=0.003; right upper panel, main effect of viral infusion, F(1,35)=19.6, *P*<0.0001 and main effect of spine type, F(2,35)=35.7, *P*<0.0001 for LA; left lower panel, main effect of viral infusion, F(1,11)=66.2, *P*<0.0001; right lower panel, main effect of viral infusion, F(1,32)=35.4, *P*<0.0001 and main effect of spine type, F(2,32)=73.8, *P*<0.0001 for BL). Similar changes were observed in the secondary branches (Figure 3c; left upper panel, main effect of viral infusion, F(1,12)=25.8, *P*=0.0004; right upper panel, main effect of viral infusion, F(1,35)=25.2, *P*<0.0001 and main effect of spine type, F(2,35)=93.6, *P*<0.0001 for LA; left lower panel, main effect of viral infusion, F(1,12)=41.02, *P*<0.0001; right lower panel, main effect of viral infusion, F(1,35)=34.3, *P*<0.0001 and main effect of spine type, F(2,35)=77.2, *P*<0.0001 for BL). These results show that GH overexpression broadly enhances spine density in the amygdala.

## Discussion

Here we characterize the impact of high levels of GH within the BLA on the level and allocation of neuronal activity during fear acquisition. Because chronic stress increases GH in the BLA and also increases fear memory strength in a GH-dependent manner,^17^ we hypothesized that GH might increase the number of neurons activated during fear encoding. In addition, because GH enhances associative plasticity,^34^ the preferential encoding of fear memory by GH-overexpressing cells could provide an additional mechanism by which GH promotes the over-encoding of fear memories. By comparing the level and allocation of neuronal activity in rats that received paired auditory fear conditioning, a well-established paradigm that produces robust long-term auditory fear memories,^21, 22, 23, 37, 38^ to that from the rats that received unpaired auditory fear conditioning, a well-established paradigm that leads to minimal associative fear memory for the tone (see Supplementary Discussion),^21, 22, 23, 36, 37, 38^ we found that high levels of GH enhance the number of amygdala neurons that are activated by fear learning, as measured by c-Fos within the amygdala after fear learning.

C-Fos demonstrates high co-localization with other IEG markers of the neuronal ensemble in the BLA following fear memory encoding and thus represents an accurate method for identifying the neurons that participate in the fear memory trace.^45, 46^ We therefore suggest that GH-mediated changes in the level and allocation of neuronal activity within the BLA reflect a role for GH in fear memory allocation and recruitment. The increase in c-Fos expression that we report was owing to a large increase in the probability of c-Fos expression by GH-overexpressing neurons, and also a more modest, yet significant, increase in the probability of c-Fos expression in neighboring uninfected neurons within the GH-overexpressing hemisphere, effects that are likely underestimated (see Supplementary Discussion). To the best of our knowledge, our data are the first to suggest that neuronal recruitment and allocation of fear memories are not static network properties. Our data also link a specific stress-associated molecular change known to produce pathological fear in both rodents^17^ to dysregulation of these properties. Because exposure to chronic stress is a risk factor for developing disorders of excessive fear,^14, 19^ such as posttraumatic stress disorder, in response to trauma, and chronic stress increases GH in the BLA, our data suggest new mechanisms by which stress promotes the excessive encoding of traumatic memories.

Here, we identify cells involved in fear memory using IEG expression following learning, rather than following long-term memory recall. This decision aligns with the many studies that have established that the cells that express IEGs during learning are also activated during long-term memory retrieval. These studies reveal that such IEG-expressing cells meet the definition of ‘engram' cells, which store a memory across days or longer and are required for the retrieval of that memory. For example, neurons ‘tagged' for c-Fos induction during learning are highly likely to express IEG products during memory recall.^10^ In addition, neurons that express c-Fos during learning are also necessary^47, 48, 49^ and sufficient^50^ for memory recall. Even in humans, neurons that are activated during the initial encoding of a stimulus are reliably activated during subsequent free recall.^51^ Thus, cells that express c-Fos during learning are a highly reliable marker for cells that participate in subsequent long-term memory retrieval.

Our data elucidate a previously unrecognized role for GH in the function of BLA neurons. Although it has long been known that the BLA synthesizes and releases GH,^52^ and the amygdala contains more GH than any other brain area outside of the pituitary^53^ (see Supplementary Discussion), virtually no studies have examined the function of locally synthesized GH within the amygdala. We show here that GH is expressed by a broad subset of excitatory and inhibitory neurons in the BLA using double fluorescent *in situ* hybridization against GH and GAD67. A comparison of GABA immunoreactivity with GAD65 and GAD67 mRNA labeling in the primate amygdala reveals a highly similar distribution of GAD67 mRNA-labeled neurons and GABA immunoreactive neurons.^54^ We cannot rule out the possibility that we did not detect a small population of GABAergic neurons that may exclusively express GAD65;^55, 56^ however, GAD67 is the primary determinant (>90%) of basal GABA levels in the brain.^57, 58^

Our study is the first to show autocrine and paracrine actions of locally synthesized GH within the BLA. In addition, the role of GH in the regulation of adult neuronal morphology is unclear. Although transgenic mice with decreased GH signaling exhibit decreased neuronal growth and decreased myelination,^59, 60^ and mice treated with GH prenatally exhibit neuronal hypertrophy as adults,^61^ these studies have several confounds. These confounds include the possibility that the role of GH is specific to early development, or that changes in peripheral GH lead to changes in insulin-like growth factor-1,^62^ which may be the critical factor in regulating neuronal morphology. Our data provide the first clear, unambiguous links between GH synthesized outside the pituitary and the regulation of dendritic spines in an adult animal.

GH-induced changes in spine density provide a potential mechanism by which neurons with high levels of GH could preferentially be recruited to a fear memory trace. The changes in spine density in the BLA that we observed following GH overexpression are similar in magnitude to those reported following chronic immobilization stress (~40% increase),^44^ and the overall spine densities we report are similar to those previously reported using three-dimensional analysis in the BLA.^28, 63, 64^ Although the specific molecular pathways in which GH may regulate dendritic spine growth remain to be elucidated, there are several pathways that may be involved in this process. For example, GH signaling pathways downstream of the GH receptor include extracellular signal-related kinase, mitogen-activated protein kinase and glycogen synthase kinase 3,^65^ all of which are implicated in the regulation of dendritic spines.^66, 67^ Furthermore, GH has been reported to regulate the actin cytoskeleton,^65^ a key component of dendritic spine growth.^66, 68^ Alternatively, GH itself can increase the transcription of c-Fos.^65, 69^ A primary factor for dendritic spine formation is neuronal activity.^66, 68^ By this model, increased c-Fos in response to increased GH may contribute to dendritic spine increases in the BLA, which in turn may predispose these BLA neurons to participate in subsequent fear memory encoding.

It is interesting to compare and contrast the impact of GH overexpression on fear memory recruitment and allocation to the impact of CREB overexpression on these properties (see Supplementary Discussion). GH and CREB are similar in that both serve as memory attractors, biasing cells to participate in memory formation (Figure 2b).^3, 70, 71^ In addition, both GH and CREB promote spine density in the BLA (Figure 3).^5^ However, there are important differences between these two factors. CREB overexpression in the BLA enhances auditory fear memory strength,^3^ but it does not change memory size, measured by the proportion of BLA neurons that express IEGs after fear memory expression in mice with overexpression of either CREB or GFP in the BLA.^3^

This finding, along with others from different neural systems,^70, 72^ have led to the idea that the recruitment of neurons is a conserved network property, even when memory strength differs.^70, 71^ In contrast, overexpression of GH in the BLA can enhance fear memory strength assessed via behavioral measures,^17^ but even when this is controlled (Figures 2a and 3a), GH overexpression enhances the number of BLA cells activated by fear memory formation (Figure 2b). One reason for these differences is that CREB is a cell-autonomous factor that remains in the nucleus of the cell in which it is expressed. In contrast, GH can be released by BLA neurons,^17, 52^ and thus may exert paracrine effects on the neighboring cells. Indeed, we found that GH overexpression increased the probability of fear conditioning-induced c-Fos expression in uninfected BLA cells (Figures 2b and c). Our results here show that neuronal recruitment is not a static network property, and suggest that high levels of GH may promote larger fear memory traces (see Supplementary Discussion).

When investigating the level and allocation of neuronal activity in the BLA, we overexpressed GH and GFP in the right hemisphere and GFP in the left hemisphere of all subjects. Although there are some studies that suggest that the right BLA is more involved in fear learning and memory than the left BLA,^73, 74^ we did not observe intrahemispheric differences in c-Fos densities in BLA sections without HSV infection following auditory fear conditioning (Figure 2d). Thus, at least with c-Fos expression, there is no evidence for lateralization of expression in the BLA after auditory fear conditioning. This means that the greater induction of c-Fos in the GH-overexpressing hemisphere that we observed after paired auditory fear conditioning (Figure 2b) is attributable to the GH overexpression, rather than lateralization of function *per se*.

Our study contributes to a substantial literature supporting the involvement of GH in cognitive function and psychiatric diseases. For example, GH deficiency has been reported to be associated with cognitive deficits, and GH replacement therapy results in moderate improvement in attention^75^ and long-term improvement in memory and mood.^76^ The studies in rats also suggest that GH treatment improves long-term memory and delays extinction in a passive avoidance test.^77^ Furthermore, the studies suggest that attention deficit hyperactivity disorder is associated with transient GH deficiency in children.^78^ Children with dwarfism have been reported to display behavioral deficits, including aggressive behavior, that are ameliorated by GH therapy.^79^ Hyperactivity of the sympathetic nervous system is associated with GH deficiency, and GH replacement therapy results in moderate improvement of this hyperactivity.^80^

GH may also be involved in reward and addiction pathways. Ghrelin triggers the secretion of GH, and ghrelin is required for alcohol reward.^81^ Ghrelin signaling in the ventral tegmental area has been shown to mediate reward-based feeding^82^ and ghrelin signaling enhances dopamine release from neurons in the ventral tegmental area,^83^ resulting in increased dopamine in multiple downstream structures including the amygdala.^84^

Collectively, our data are the first to show that neuronal network activation in response to fear conditioning is a dynamic network property, and we are also the first to link a specific stress-associated molecule^17^ to dysregulation of this property. It is of great interest to determine whether GH, which is broadly expressed by neurons in the adult brain, biases memory allocation in other neural circuits. It is also of interest to determine sex differences in GH expression and the function of GH within the BLA (see Supplementary Discussion). Finally, it will be important to examine the expression of GH in the human amygdala in clinical populations, such as patients with posttraumatic stress disorder.
