# Supplementary material for: Growth hormone biases amygdala network activation after fear learning
Source: Transl Psychiatry. 2016 Nov 29;6(11):e960–. doi: 10.1038/tp.2016.203 (PMC5290350; doi:10.1038/tp.2016.203)
Supplement: Supplementary Information [file tp2016203x1.pdf]

SUPPLEMENTARY MATERIALS

Supplementary Materials and Methods……………………p. 2-8

Supplementary Results…………………………………….p.9-10

Supplementary Discussion………………………………..p.10-13

Supplementary References……………………………….p.14-18

Supplementary Figure Legends…………………………...p.19-20

SUPPLEMENTARY MATERIALS AND METHODS

Experimental Subjects

All experiments used individually housed male rats (68-72°F; 12-h light/dark cycle, 7AM lights on). All procedures were in accordance with the US National Institutes of Health (NIH) Guide for the Care and Use of Laboratory Animals.

Virus

Amplicon construction

The rat presomatotropin (growth hormone) gene was expressed using a bicistronic HSV-based promoter, which simultaneously drives expression of the presomatotropin gene from the α-4 promoter and enhanced green florescent protein (eGFP) from the α-22 promoter. These promoters are derived from the HSV genome and are commonly used in HSV amplicons to drive gene expression. An amplicon expressing only eGFP from the α-22 promoter was used as a control. Using these HSV amplicons, the peak of transgene expression occurs approximately 4 days after infusion but continues to express for at least a week [1, 2], a time point that coincides with our experimental manipulations and time of sacrifice. These amplicons were cloned previously in our laboratory [3] into the HSV backbone pα22-eGFP [4].

Virus preparation

Virus was generated using standard methods [5] and was made by the Viral Core at the McGovern Institute for Brain Research (Cambridge, MA, USA). Briefly, endotoxin free DNA,

which was transfected into 2–2 cells was generated through plasmid amplification. Following plasmid amplification, cells were superinfected with 5dl1.2 helper virus. After two days, cells were frozen and thawed three times, sonicated to release infectious viral particles, and centrifuged to clear the medium of cell debris. A supernatant resulting from this process was in turn passaged twice onto 2–2 cells. After sonication and centrifugation, the supernatant was purified on a sucrose gradient, pelleted, and resuspended in 10% sucrose in Dulbecco's Phosphate-Buffered Saline (D-PBS).

Aliquots of each amplicon were stored at −80°C until use. Amplicon titers ranged from 1 to 3 x 10^8/ml. Within each experiment, control and GH-expressing viral titers were diluted to be identical.

Stereotaxic Surgery

Male rats used for c-Fos quantification experiments underwent handling for four days prior surgery and for four days afterwards in order to minimize baseline levels of c-Fos expression. Rats were anesthetized (with either Nembutal at 65 mg/kg, or a ketamine:xylazine:acepromazine cocktail at 100:100:10 mg/kg, i.p.) and mounted in a stereotaxic frame. Lambda and bregma were placed in the same plane. Small holes were drilled for intra-cranial placement of the injector within the BLA (A/P -2.4 mm, M/L +/-5.1mm, D/V -7.0 mm) relative to brain surface and bregma. These coordinates were derived from a stereotaxic brain atlas [6]. Rats received intra-BLA injections of HSV amplicons to express GH and GFP (the experimental hemisphere) or only GFP (the control hemisphere). Each rat received two infusions of virus into the BLA of each hemisphere (1µl: A/P -2.4, M/L +/-5.1, D/V -7.2, and 1µl: A/P -

2.4, M/L +/-5.1, D/V -6.9) for a total of 2 µl per BLA.

Virus was infused with Nanofil 35 gauge stainless steel bevel needles (catalog # NF35BV, World Precision Instruments, Inc., Sarasota, FL) attached to a 10µl Nanofil syringe (Hamilton Company, Reno, NV). Hamilton syringes were mounted in a stereotaxic barrel holder, and the rate of virus delivery was controlled by a syringe pump (Harvard Apparatus, Holliston, MA). Virus was infused at 0.1 µl/min for 10 min at each infusion site (2 µl total volume per hemisphere). Injectors remained in the brain for 10 min before being raised for the second infusion, and for 10 min before being slowly withdrawn. Incisions were closed with wound clips and Ketoprofen (1 mg/ml/kg b.w., s.c.) was administered for pain and inflammation.

In Situ Hybridization

Naïve rats (n = 3), who had not experienced surgical procedures or behavioral testing, were anesthetized (Isoflurane; 3%) and transcardially perfused with ice-cold saline containing 0.1% diethylpyrocarbonate (DEPC), followed by an ice-cold fixative containing 4% paraformaldehyde and 0.1% DEPC in 0.1M PBS, pH 7.3. Brains were immediately removed and post-fixed in the same fixative for 2 hr. The brains were then cryoprotected in 30% sucrose in 0.1M PBS (72 hr at 4°C) and cryosectioned (-23°C) into sixteen 20-µm thick sections containing the middle BLA (between -2.8 to -3.14 mm posterior to bregma). The fluorescence in-situ hybridization staining was carried out using the QuantiGene ViewRNA ISH Tissue 2-Plex kit (Affymetrix; Santa Clara, CA) and staining procedures were performed according to their protocol. Tissue sections were first dehydrated using increasing concentration of ethanol (50%, 70% and 100%, 10 min for each dehydration step) and then baked at 60°C for 30 min. The

sections were then treated with Proteinase solution at 40°C for 40 min and hybridized for 2 hours at 40°C with custom-designed QuantiGene ViewRNA probes to complement GH and GAD67. Unbound probes were washed out with Wash buffer and the bound probes were then hybridized with PreAmp solution for 25 min at 40° C, followed by Amp solution for 15 min at 40° C. Two Label Probes (LP), conjugated to alkaline phosphatase (AP), were used to visualize the hybridization, of which the GH was visualized by LP-AP-type 1 that reacted with Fast Red Substrate to deliver Cy3 fluorescence, whereas GAD67 by LP-AP-type 6 that reacted with Fast Blue Substrate to deliver Cy5 fluorescence. Sections were counterstained with Gill's Hematoxylin (Sigma-Aldrich; St. Louis, MO) for the anatomical localization of amygdala nuclei, and with DAPI to label the nuclei. Slides were then mounted in Ultramount mounting medium (DAKO; Carpinteria, CA).

We ran GH and GAD67 negative controls, in which sections (one slide with 2 sections) containing the BLA were processed as described above, but no probe against GH or GAD67 was added. These controls revealed the complete absence of red puncta when the GH probe was omitted, and the complete absence of green puncta when the GAD67 probe was omitted. In addition, as a positive control to test the RNA integrity, we used a Rat Hprt probe (type 6, Catalog Number VC6- 14226-01, Lot# 112002913, Affymetrix, Santa Clara, CA) in lieu of the GH or GAD67 probes for one slide (containing two sections). One Hprt control slide was run with each group of slides being processed. These controls revealed strong labeling of the Hprt mRNA throughout the tissue (Supp. Fig. 4).

Microscopy and Data Analyses

For Dendritic Spine Quantification

Confocal microscopy images were analyzed using Neurolucida software with Autospine (Neurolucida version 10) to measure spine density in apical branches of BL neurons. Spines were placed in one of three categories: thin, mushroom, and stubby. These stubby spines were identified by the absence of a neck [7] and newly formed spines were identified as thin, with long necks and small bulbous heads [7-9]. Thin spines are the most numerous type of spines, and are thought to be the most plastic spine type [7, 10-12]. In contrast, mushroom spines, which were identified by a large, mushroom-shaped head [13], are believed to represent a more stable synaptic structure that follows the induction of plasticity, and are less numerous [14-19]. Importantly, these mushroom-shaped spines may be more effective excitatory synaptic sites, as their heads contain larger numbers of glutamate receptors, which may contribute to increased synaptic strength, serving as a locus of a stable circuit for long-term fear memory [20, 21]. Thin spines, in comparison, have smaller heads containing fewer glutamate receptors [16], and are thought to be capable of expanding and stabilizing or retracting, properties that are associated with new learning [12, 13, 16, 22]. In addition to spine head size, spine neck length also plays a major role in spine function [23-26]. The length of spine neck reduces, and the width increases, following the induction of long-term potentiation (LTP) [23, 24], and spines with shorter necks have larger excitatory post-synaptic potentials than spines with longer necks [25, 26], suggesting that increased numbers of stubby spines after the induction of LTP contribute to stronger post-synaptic potentials.

To get a more accurate quantification of spine density, we used three-dimensional dendritic analysis combined with confocal imaging; this enabled the visualization of spines pointing directly toward the surface or extending beneath the dendrite [27]. Such methodology thus

results in higher spine densities than are observed with Golgi staining [27-30]. All spine analysis was performed by at least two observers who manually classified and counted each spine. All observers were blinded to the conditions of the images being analyzed.

Images of 40 µm-thick BLA sections were acquired, with *z*-step 0.25 µm, using a 60x oil-immersion objective (numerical aperture 1.4; pixel size, 0.23 x 0.23 µm) and an Olympus FluoView FV1000 confocal microscope. Images consisting of 10µm of dendritic branch length starting 20µm from the cell body were used to analyze spine density in the primary branches, and 10µm sections were imaged from dendrites branching off the primary branches to analyze spine density in secondary branches. Such an approach has been previously used in the BLA [31-33].

Statistics

The number of animals (n) in the experimental and control groups for each experiment are reported in the figure legends. For all statistical tests, the significance threshold was $p \leq 0.05$.

Freezing behavior was defined as periods of at least 1 s with the complete absence of movement except breathing; it was measured with manual scoring. The percent of time spent freezing during intervals of interest was quantified, and these results were analyzed using analysis of variance (ANOVA). Post hoc Fisher's PLSD tests were performed after a significant omnibus F-ratio.

To analyze the effects of GH on memory size and allocation (Fig. 2) and dendritic spine density (Fig. 3), the average value for each rat was computed for each measure of interest. Thus, statistical significance was assessed using degrees of freedom based on the number of animals, rather than the number of cells, to avoid inappropriate distortion of the statistical significance.

ANOVAs were performed on each measure of interest. The factors examined included Viral Infusion (GFP and GH), Cell population (Infected and Uninfected), Region (LA and BL), Conditioning (Paired and Unpaired), and Spine type (thin, mushroom, and stubby).

To determine whether the percentage of c-Fos expression in infected cells in the GH-overexpressing hemisphere was greater than chance (Fig. 3a), the factor by which c-Fos expression in the GH-overexpressing hemisphere exceeded c-Fos expression in the GFP-overexpressing hemisphere was calculated. This was 1.90 for the LA and 1.76 for the BL. The percentage of the uninfected and infected cells expressing c-Fos in the GFP-overexpressing hemisphere was multiplied by this factor to generate the expected percentages of cells expressing c-Fos in the GH hemisphere, assuming c-Fos was randomly distributed across the infected and uninfected cells of the GH-overexpressing hemisphere. A one-tailed, one-sample t-test was used to test the specific hypotheses that the percentages of infected or uninfected cells in the GH-overexpressing hemisphere were greater than chance levels.

For *in situ* hybridization, percentages of cells expressing GAD67 were calculated by dividing the total number of GAD67+ cells per region over the total number of all DAPI+ nuclei per region. Similarly, percentages of cells expressing GH were calculated by dividing the total numbers of GH cells by the total numbers of DAPI+ nuclei. The percentage of GH cells expressing GAD67 was calculated by dividing the total number of GH+/GAD67+ cells by the total number of GH+ cells. A one-tailed, one-sample t-test was used to determine whether the average percent of GAD67+/GH+ double-positive cells within each brain region was significantly higher than the mean percentage of GH+ cells within the same brain region.

SUPPLEMENTARY RESULTS

GH overexpression does not impact the retrieval of an established fear memory or the acquisition of extinction memory

Rats received auditory fear conditioning. Two days later, rats received intra-BLA bilateral infusions of either the GH-expressing virus or the GFP-expressing virus. After recovery, animals received context fear extinction and two days of auditory fear extinction. The GH- and GFP-overexpressing groups did not differ within any session (Supp. Fig. 1) (Fear Conditioning: main effect of group: $F(1,6) = 0.01$, $p = 0.92$; Context Extinction: main effect of group: $F(1,6) = 0.03$, $p = 0.87$; Auditory Fear Extinction Training: main effect of group: $F(1,6) = 0.04$, $p = 0.85$; Auditory Fear Extinction Recall: main effect of group: $F(1,6) = 0.38$, $p = 0.56$).

Cell density and viral infection were comparable between the GH and GFP hemispheres

After six days of recovery, animals received either paired or unpaired auditory fear conditioning, and were sacrificed for immunohistochemistry one hour after fear conditioning (Supp. Fig. 1b)

Significant and similar increases of freezing were observed in the Paired and Unpaired groups during fear conditioning (Supp. Fig. 1c)(main effect of Trial: $F(2,29) = 13.1$, $p < 0.0001$; Conditioning type X Trial interaction: $F(2,29) = 0.15$, $p = 0.86$), reflecting an amalgam of freezing triggered by the context and tone, and non-associative sensitization to the shock. It is important to note that this freezing does not reflect the robust differential long-term auditory fear levels that are reliably elicited by paired (strong auditory fear memory) versus unpaired (weak

auditory fear memory) auditory fear conditioning [34-36]. The use of the Unpaired control group enabled us to determine the level of c-Fos induction that was related to the sensory perception of shock, tone, and context, rather than the induction of long-term associative plasticity [present only in animals that receive Paired fear conditioning [34]].

In addition to using anatomical features to show that the coronal brain sections we analyzed were comparable between the GH and GFP groups, we examined both cell density and viral infection. Immunohistochemistry from sections containing the LA and BL reveals that the cell density did not differ between the GFP and GH groups (main effect of Viral infusion: $F(1,40) = 0.82$, $p = 0.37$)(Supp. Fig. 1d, left). Additionally, the rate of viral infection did not differ between the GFP and GH groups (main effect of Viral infusion: $F(1,40) = 0.06$, $p = 0.81$), or between Paired and Unpaired groups (main effect of Conditioning type: $F(1,40) = 0.76$, $p = 0.39$) (Supp. Fig. 1e).

SUPPLEMENTARY DISCUSSION

Our use of an unpaired control group provided two important controls. Unpaired auditory fear conditioning leads to minimal long-term auditory fear memory in previously published reports [37], and we show here that neither conditioning-induced c-Fos expression nor the allocation of c-Fos across BLA neurons is impacted by GH overexpression after unpaired auditory fear conditioning. Thus, our results are most consistent with the idea that GH regulates plasticity [38]. In addition, because c-Fos is not increased in the GH-overexpressing hemisphere relative to the GFP-overexpressing hemisphere in rats that received unpaired conditioning (Fig. 2e), this shows that GH overexpression is not sufficient to enhance translation of c-Fos protein.

It is likely that our methods underestimate the impact of GH on BLA function. We preferentially selected brain sections for immunohistochemistry in which the infection rate was low (~3.5%; Supp. Fig. 1d). Higher levels of infection prevented architectural resolution of individual dendritic branches, and also led to saturation of the florescent signal. Although we found that GH-overexpressing cells were strongly biased to express c-Fos after associative fear learning (~70% of GH-overexpressing cells also expressed c-Fos, whereas only ~20% of uninfected neighboring cells expressed c-Fos), because these comprise only a small percentage of the overall number of cells in our selected sections, we found that the majority of c-Fos was still expressed by uninfected neighbor cells. However, we did observe a moderate and significant increase in c-Fos expression in the uninfected neighbor cells, providing strong support for the idea that GH exerts paracrine influences within the BLA. It is likely that in sections with higher infection rates, a significantly higher proportion of total c-Fos expression would be observed in the GH-overexpressing cells.

A second way in which the effect size of GH may be underestimated is by our use of GH- and GFP-overexpression within the opposing hemispheres of single animals. This design allowed us to eliminate GH-induced changes in behavior as a potential experimental confound. However, it is possible that GH overexpression in the BLA of one hemisphere could enhance the function of the contralateral BLA, either by enhancing fear behavior, or by enhancing fear learning in networks spanning the two hemispheres. Despite this constraint, we still observed significant changes in the level and allocation of neuronal activity in both the LA and BL of the GH-overexpressing hemisphere.

A final way in which we may be underestimating the impact of GH on the BLA concerns our use of HSV to overexpress GH. We have previously reported that chronic stress increases GH in the BLA by approximately 200% [3]. We have also found that the use of these amplicons in the hippocampus increases GH by approximately 100% over endogenous levels [39], which are lower than that in the BLA. Thus, our overexpression of GH in the BLA likely does not quite reach the levels observed following chronic stress.

GH is synthesized by other brain regions outside of the pituitary, including the hippocampus. In the hippocampus, GH is increased following memory formation [40], and it also regulates both neurogenesis [41] and long-term potentiation [38]. It is also downregulated in the hippocampus following prolonged stress, and rescue of this decrease restores normal hippocampal function [39]. Thus, GH may make an important contribution to “normal” function in the adult brain, and decreases in GH within specific brain regions may contribute to impaired function within those regions.

Surprisingly, we observed that viral transduction of BLA cells with HSV expressing GFP alone increased c-Fos expression (Fig. 2b,e, right panels). This effect cannot be attributed to bleeding of the green GFP signal into the red c-Fos channel (Supp. Fig. 3), and it was observed in both the Paired and Unpaired fear conditioning groups. Thus, HSV viral transduction increases c-Fos expression in a manner that is unrelated to the formation of a tone-shock association. While this suggests caution in extending HSV gene therapies to human applications, the induction of c-Fos by HSV transduction did not preclude our ability to contrast conditioning-induced gene expression in the hemispheres expressing GFP versus the hemispheres expressing GFP and GH.

Constraints on the size of a fear memory in the BLA are thought to arise through a competitive process derived from intrinsic cell excitability and disynaptic inhibition between principal neurons [42-44]. It is possible that viral GH overexpression disrupts this lateral competition within the BLA, permitting broader expression of immediate early genes across BLA cells. Indeed, because we observed GH expression within GAD67+ BLA interneurons (Fig. 1), it is possible that GH overexpression dysregulates inhibitory neurotransmission within the BLA. An alternative is that the viral overexpression GH may, through paracrine actions on neighboring BLA neurons, either trigger an upregulation of endogenous GH in these cells, or induce biological changes, such as increased spine density or changes in gene transcription, which lead to enhanced recruitment of these cells into the fear memory trace. Future studies will undoubtedly shed light on the specific mechanism by which paracrine actions of GH within the BLA impact the function of neighboring neurons in fear memory.

While our study was performed in male rats, there are reports of sex differences in GH expression, as well as its regulation by short-term stress, in the hippocampus [45]. In addition, there are well-known sex differences in fear conditioning [46-48], the impact of stress on learning mediated by the amygdala [49], and on the regulation of dendritic spines by stress [50, 51].. Sex differences in GH in the BLA, and the regulation of this by stress, remain completely unexplored. These are all important topics for future studies.

SUPPLEMENTARY REFERENCES

1. Carlezon WA, Jr., Thome J, Olson VG, Lane-Ladd SB, Brodkin ES, Hiroi N, *et al*. Regulation of cocaine reward by CREB. *Science* 1998; **282**(5397)**:** 2272-2275.

2. Lim F, Hartley D, Starr P, Lang P, Song S, Yu L, *et al*. Generation of high-titer defective HSV-1 vectors using an IE 2 deletion mutant and quantitative study of expression in cultured cortical cells. *BioTechniques* 1996; **20**(3).

3. Meyer RM, Burgos-Robles A, Liu E, Correia SS, Goosens KA. A ghrelin-growth hormone axis drives stress-induced vulnerability to enhanced fear. *Mol Psychiatry* 2014; **19**(12)**:** 1284-1294.

4. Kaufer D, Ogle WO, Pincus ZS, Clark KL, Nicholas AC, Dinkel KM, *et al*. Restructuring the neuronal stress response with anti-glucocorticoid gene delivery. *Nat Neurosci* 2004; **7**(9)**:** 947-953.

5. Lim F, Neve RL. Generation of high-titer defective HSV-1 vectors. *Current protocols in neuroscience / editorial board, Jacqueline N Crawley [et al]* 2001; **Chapter 4:** Unit 4 13.

6. Paxinos G, Watson C. *The Rat Brain in Stereotaxic Coordinates-The New Coronal Set*, vol. 5th Edition. Elsevier Academic Press, 2005.

7. Peters A, Kaiserman-Abramof IR. The small pyramidal neuron of the rat cerebral cortex. The perikaryon, dendrites and spines. *The American journal of anatomy* 1970; **127**(4)**:** 321-355.

8. Tashiro A, Yuste R. Structure and molecular organization of dendritic spines. *Histology and histopathology* 2003; **18**(2)**:** 617-634.

9. Arellano JI, Benavides-Piccione R, Defelipe J, Yuste R. Ultrastructure of dendritic spines: correlation between synaptic and spine morphologies. *Frontiers in neuroscience* 2007; **1**(1)**:** 131-143.

10. Harris KM, Jensen FE, Tsao B. Three-dimensional structure of dendritic spines and synapses in rat hippocampus (CA1) at postnatal day 15 and adult ages: implications for the maturation of synaptic physiology and long-term potentiation. *J Neurosci* 1992; **12**(7)**:** 2685-2705.

11. Holtmaat AJ, Trachtenberg JT, Wilbrecht L, Shepherd GM, Zhang X, Knott GW, *et al*. Transient and persistent dendritic spines in the neocortex in vivo. *Neuron* 2005; **45**(2)**:** 279-291.

12. Zuo Y, Lin A, Chang P, Gan WB. Development of long-term dendritic spine stability in diverse regions of cerebral cortex. *Neuron* 2005; **46**(2)**:** 181-189.

13. Dumitriu D, Hao J, Hara Y, Kaufmann J, Janssen WG, Lou W, *et al.* Selective changes in thin spine density and morphology in monkey prefrontal cortex correlate with aging-related cognitive impairment. *J Neurosci* 2010; **30**(22)**:** 7507-7515.

14. Yoshihara Y, De Roo M, Muller D. Dendritic spine formation and stabilization. *Curr Opin Neurobiol* 2009; **19**(2)**:** 146-153.

15. Bourne JN, Harris KM. Balancing structure and function at hippocampal dendritic spines. *Annual review of neuroscience* 2008; **31:** 47-67.

16. Matsuzaki M, Honkura N, Ellis-Davies GC, Kasai H. Structural basis of long-term potentiation in single dendritic spines. *Nature* 2004; **429**(6993)**:** 761-766.

17. Harvey CD, Svoboda K. Locally dynamic synaptic learning rules in pyramidal neuron dendrites. *Nature* 2007; **450**(7173)**:** 1195-1200.

18. Sakurai E, Hashikawa T, Yoshihara Y, Kaneko S, Satoh M, Mori K. Involvement of dendritic adhesion molecule telencephalin in hippocampal long-term potentiation. *Neuroreport* 1998; **9**(5)**:** 881-886.

19. Kopec CD, Real E, Kessels HW, Malinow R. GluR1 links structural and functional plasticity at excitatory synapses. *J Neurosci* 2007; **27**(50)**:** 13706-13718.

20. Kasai H, Matsuzaki M, Noguchi J, Yasumatsu N, Nakahara H. Structure-stability-function relationships of dendritic spines. *Trends Neurosci* 2003; **26**(7)**:** 360-368.

21. Kasai H, Fukuda M, Watanabe S, Hayashi-Takagi A, Noguchi J. Structural dynamics of dendritic spines in memory and cognition. *Trends Neurosci* 2010; **33**(3)**:** 121-129.

22. Holtmaat A, Wilbrecht L, Knott GW, Welker E, Svoboda K. Experience-dependent and cell-type-specific spine growth in the neocortex. *Nature* 2006; **441**(7096)**:** 979-983.

23. Fifkova E, Anderson CL. Stimulation-induced changes in dimensions of stalks of dendritic spines in the dentate molecular layer. *Exp Neurol* 1981; **74**(2)**:** 621-627.

24. Fifkova E, Van Harreveld A. Long-lasting morphological changes in dendritic spines of dentate granular cells following stimulation of the entorhinal area. *J Neurocytol* 1977; **6**(2)**:** 211-230.

25. Araya R, Jiang J, Eisenthal KB, Yuste R. The spine neck filters membrane potentials. *Proc Natl Acad Sci U S A* 2006; **103**(47)**:** 17961-17966.

26. Araya R, Vogels TP, Yuste R. Activity-dependent dendritic spine neck changes are correlated with synaptic strength. *Proc Natl Acad Sci U S A* 2014; **111**(28)**:** E2895-2904.

27. Ryan SJ, Ehrlich DE, Rainnie DG. Morphology and dendritic maturation of developing principal neurons in the rat basolateral amygdala. *Brain Struct Funct* 2014.

28. Trommald M, Jensen V, Andersen P. Analysis of dendritic spines in rat CA1 pyramidal cells intracellularly filled with a fluorescent dye. *J Comp Neurol* 1995; **353**(2)**:** 260-274.

29. Feldman ML, Peters A. A technique for estimating total spine numbers on Golgi-impregnated dendrites. *J Comp Neurol* 1979; **188**(4)**:** 527-542.

30. Trommald M, Hulleberg G. Dimensions and density of dendritic spines from rat dentate granule cells based on reconstructions from serial electron micrographs. *J Comp Neurol* 1997; **377**(1)**:** 15-28.

31. Bennur S, Shankaranarayana Rao BS, Pawlak R, Strickland S, McEwen BS, Chattarji S. Stress-induced spine loss in the medial amygdala is mediated by tissue-plasminogen activator. *Neuroscience* 2007; **144**(1)**:** 8-16.

32. Gourley SL, Swanson AM, Koleske AJ. Corticosteroid-induced neural remodeling predicts behavioral vulnerability and resilience. *J Neurosci* 2013; **33**(7)**:** 3107-3112.

33. Shansky RM, Hamo C, Hof PR, Lou W, McEwen BS, Morrison JH. Estrogen promotes stress sensitivity in a prefrontal cortex-amygdala pathway. *Cereb Cortex* 2010; **20**(11)**:** 2560-2567.

34. Maren S. Auditory fear conditioning increases CS-elicited spike firing in lateral amygdala neurons even after extensive overtraining. *Eur J Neurosci* 2000; **12**(11)**:** 4047-4054.

35. Curzon P, Rustay N, Browman K. Cued and Contextual Fear Conditioning for Rodents. In: JJ B (ed). *Methods of Behavior Analysis in Neuroscience. 2nd edition.* CRC Press/Taylor & Francis: Boca Raton (FL), 2009.

36. Schafe GE, Nadel NV, Sullivan GM, Harris A, LeDoux JE. Memory consolidation for contextual and auditory fear conditioning is dependent on protein synthesis, PKA, and MAP kinase. *Learn Mem* 1999; **6**(2)**:** 97-110.

37. Rogan MT, Staubli UV, LeDoux JE. Fear conditioning induces associative long-term potentiation in the amygdala. *Nature* 1997; **390**(6660)**:** 604-607.

38. Zearfoss NR, Alarcon JM, Trifilieff P, Kandel E, Richter JD. A molecular circuit composed of CPEB-1 and c-Jun controls growth hormone-mediated synaptic plasticity in the mouse hippocampus. *J Neurosci* 2008; **28**(34)**:** 8502-8509.

39. Vander Weele CM, Saenz C, Yao J, Correia SS, Goosens KA. Restoration of hippocampal growth hormone reverses stress-induced hippocampal impairment. *Front Behav Neurosci* 2013; **7:** 66.

40. Donahue CP, Jensen RV, Ochiishi T, Eisenstein I, Zhao M, Shors T, *et al*. Transcriptional profiling reveals regulated genes in the hippocampus during memory formation. *Hippocampus* 2002; **12**(6)**:** 821-833.

41. Devesa P, Agasse F, Xapelli S, Almenglo C, Devesa J, Malva JO, *et al*. Growth hormone pathways signaling for cell proliferation and survival in hippocampal neural precursors from postnatal mice. *BMC Neurosci* 2014; **15:** 100.

42. Han JH, Kushner SA, Yiu AP, Cole CJ, Matynia A, Brown RA, *et al*. Neuronal competition and selection during memory formation. *Science* 2007; **316**(5823)**:** 457-460.

43. Kim J, Kwon JT, Kim HS, Han JH. CREB and neuronal selection for memory trace. *Frontiers in neural circuits* 2013; **7:** 44.

44. Yiu AP, Mercaldo V, Yan C, Richards B, Rashid AJ, Hsiang HL, *et al*. Neurons are recruited to a memory trace based on relative neuronal excitability immediately before training. *Neuron* 2014; **83**(3)**:** 722-735.

45. Donahue CP, Kosik KS, Shors TJ. Growth hormone is produced within the hippocampus where it responds to age, sex, and stress. *Proc Natl Acad Sci U S A* 2006; **103**(15)**:** 6031-6036.

46. Maren S, De Oca B, Fanselow MS. Sex differences in hippocampal long-term potentiation (LTP) and Pavlovian fear conditioning in rats: positive correlation between LTP and contextual learning. *Brain Res* 1994; **661**(1-2)**:** 25-34.

47. Dalla C, Shors TJ. Sex differences in learning processes of classical and operant conditioning. *Physiol Behav* 2009; **97**(2)**:** 229-238.

48. Cover KK, Maeng LY, Lebron-Milad K, Milad MR. Mechanisms of estradiol in fear circuitry: implications for sex differences in psychopathology. *Transl Psychiatry* 2014; **4:** e422.

49. Waddell J, Bangasser DA, Shors TJ. The basolateral nucleus of the amygdala is necessary to induce the opposing effects of stressful experience on learning in males and females. *J Neurosci* 2008; **28**(20)**:** 5290-5294.

50. Shors TJ, Chua C, Falduto J. Sex differences and opposite effects of stress on dendritic spine density in the male versus female hippocampus. *J Neurosci* 2001; **21**(16)**:** 6292-6297.

51. Shors TJ, Falduto J, Leuner B. The opposite effects of stress on dendritic spines in male vs. female rats are NMDA receptor-dependent. *Eur J Neurosci* 2004; **19**(1)**:** 145-150.

SUPPLEMENTARY FIGURE LEGENDS

Supplementary Figure 1. Growth hormone overexpression does not impact fear memory recall or extinction. Rats received auditory fear conditioning. Two days later, rats received intra-BLA infusion of either GH-expressing or GFP-expressing HSV amplicons. Six days later, rats received contextual extinction and auditory extinction training. Auditory extinction recall was conducted one day later. Conditional freezing for all the sessions is depicted. Freezing during auditory extinction training is depicted as blocks; each block is the average freezing across two sequential tone presentations. All error bars indicate mean ± s.e.m.

Supplementary Figure 2. Local cell density and infection rate do not differ across the GH-overexpressing and GFP-overexpressing hemispheres. (**a**) Bright-field/fluorescence (GFP) images in the amygdala at low magnification (2.5X) showing HSV spread. Right image shows a coronal rat brain section with an HSV amplicon co-expressing GH and GFP infused into the right amygdala, and an HSV amplicon expressing GFP only infused into the left amygdala (left panel). Scale bars: 250μm. (**b**) Triple fluorescent immunostaining was performed to visualize GFP (green), c-Fos (red), and nuclei (DAPI, blue) in brain sections containing the LA and BL, and confocal images were acquired for data analysis. White arrows indicate the GFP+ and c-Fos+ nuclei in the green and red channels. Yellow arrows indicate GFP/c-Fos double-positive nuclei in the merged image. (**c**) Paired and unpaired fear conditioning protocols elicited similar levels of freezing behavior during conditioning. (**d**) Cell density, calculated as the number of DAPI+ cells in the volume of the target brain region, did not differ between the GH and GFP conditions, nor between the Paired and Unpaired conditions. (**e**) The percentage of cells infected with the virus did not differ between the GH and GFP conditions, or between the Paired and Unpaired conditions. Scale bars represent 36.1 μm. For each measure, an average value was computed for the GH hemisphere and a second average was calculated for the GFP hemisphere for each animal. All statistical tests were computed using these per-animal averages. All error bars indicate mean ± s.e.m.

Supplementary Figure 3. No cross-talk was observed between the green and red fluorescence channels for c-Fos immunostaining of the BLA. Sections with c-Fos immunostaining do not show any crossover of green and red fluorescence emission. The green arrows indicate infected (GFP-overexpressing) cell bodies which lack signal in the red channel. Similarly, red arrows indicate c-Fos expressing cell bodies which lack signal in the green channel.

Supplementary Figure 4. In situ hybridization against Hypoxanthine Phosphoribosyltransferase 1 (Hprt). For some brain sections containing LA and BL, *in situ* hybridization was performed with a probe to target Hprt (positive control); all other steps were identical to those used for other sections. We observed strong Hprt staining throughout the BLA for each batch of slides with this method.
