# Supplementary figures and images for: Growth hormone biases amygdala network activation after fear learning
Source: Transl Psychiatry. 2016 Nov 29;6(11):e960–. doi: 10.1038/tp.2016.203 (PMC5290350; doi:10.1038/tp.2016.203)

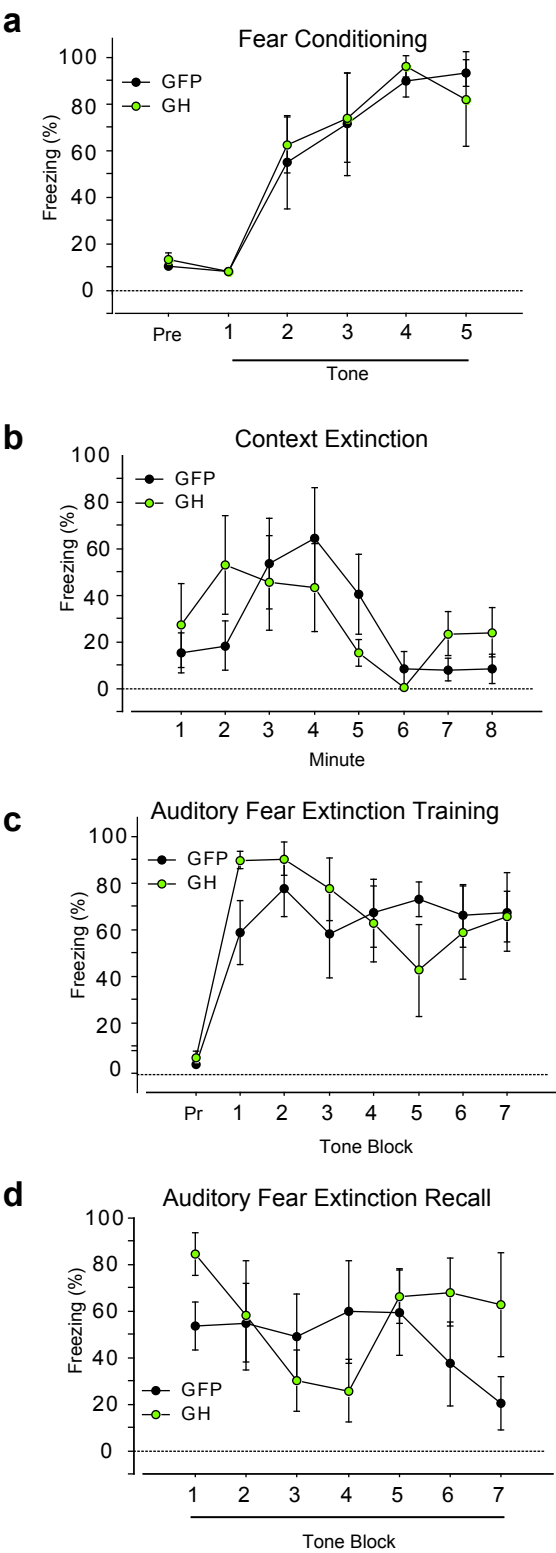

Supplement: Supplementary Figure 1 [file tp2016203x2.pdf]

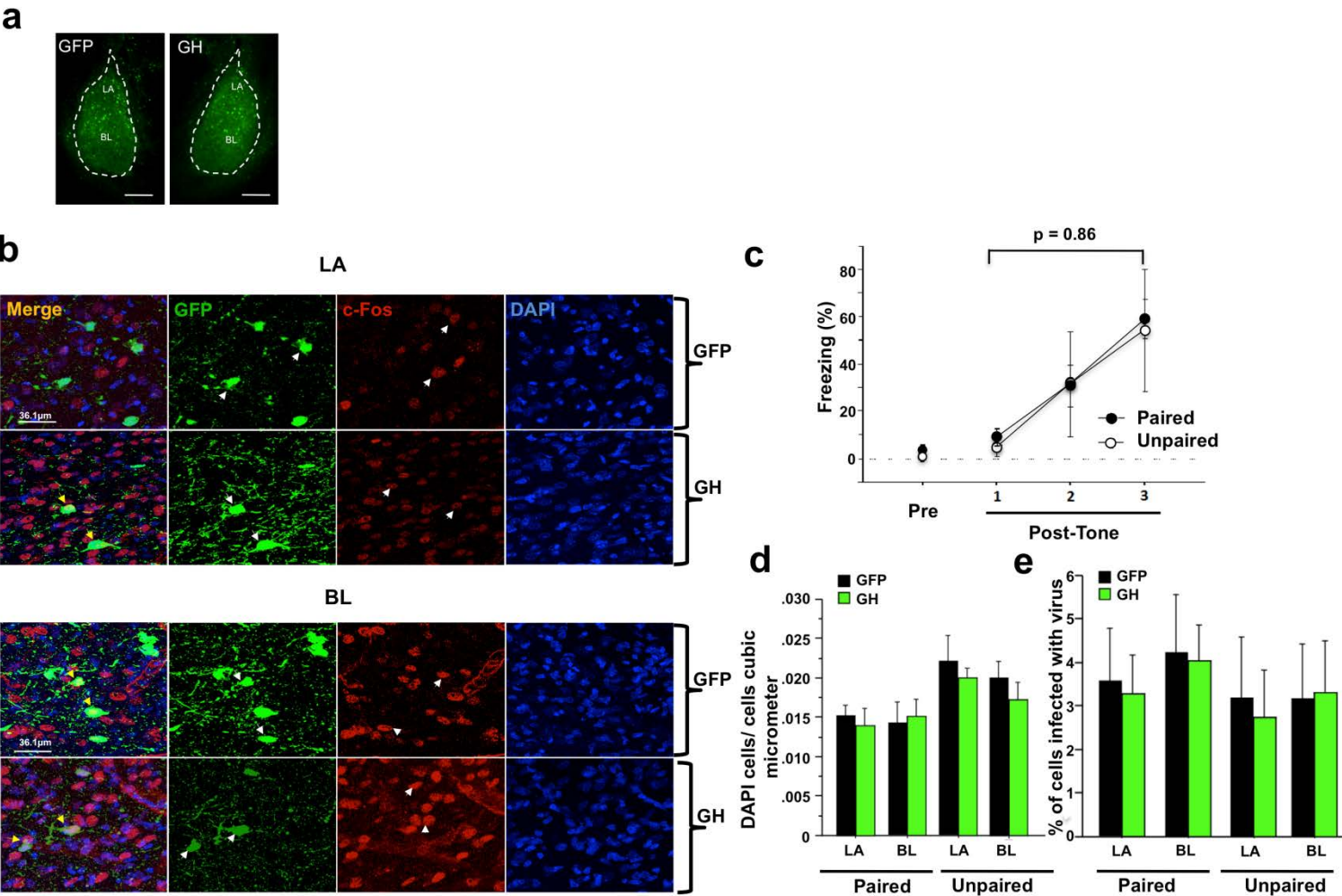

Supplement: Supplementary Figure 2 [file tp2016203x3.pdf]

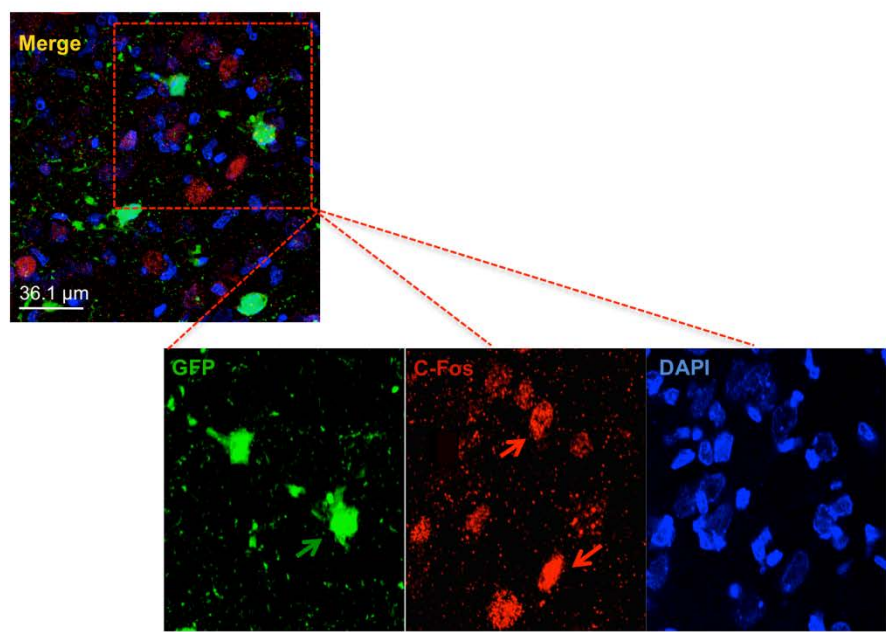

Supplement: Supplementary Figure 3 [file tp2016203x4.pdf]

### Rat Hprt Probe Positive Control

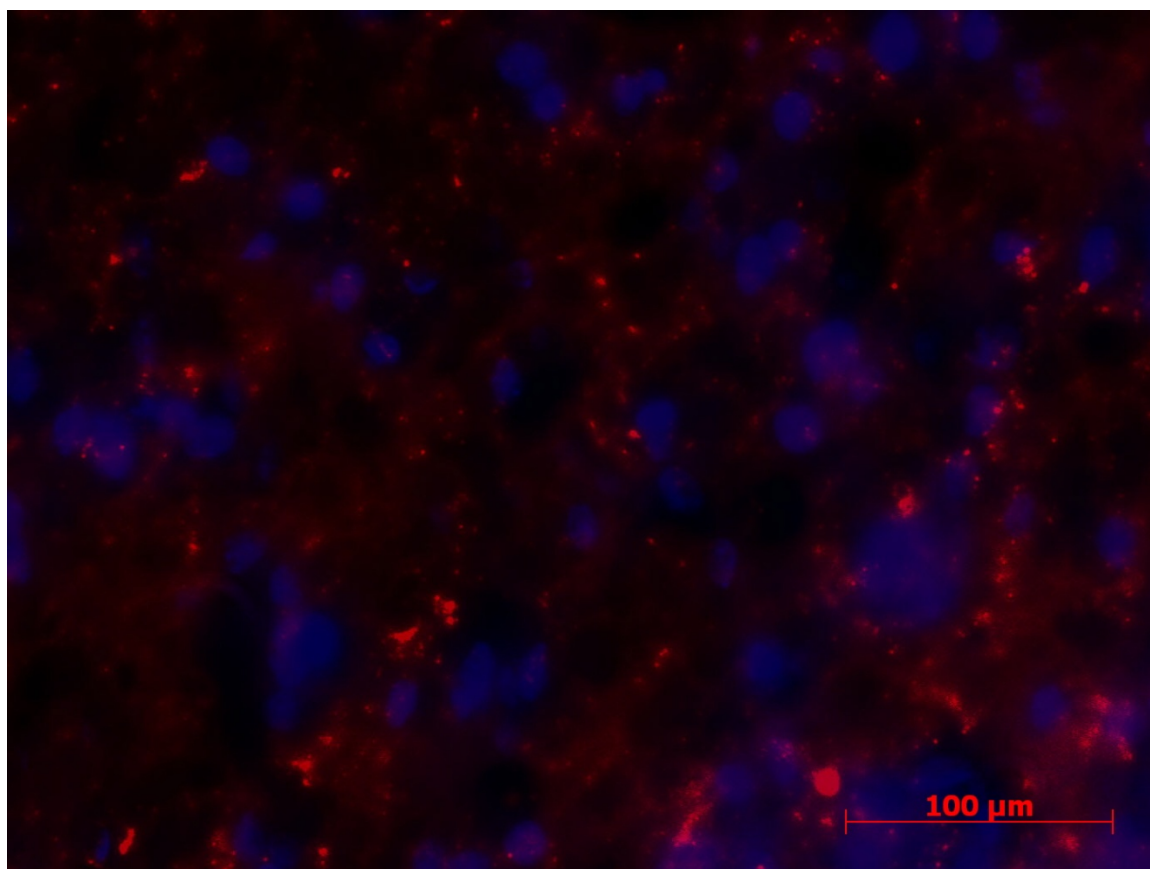

Supplement: Supplementary Figure 4 [file tp2016203x5.pdf]
